# Construction and validation of a nomogram prediction model for chronic low back pain after PKP for lumbar compression fractures

**DOI:** 10.1097/MD.0000000000034752

**Published:** 2023-10-27

**Authors:** Guang-hua Deng

**Affiliations:** a Ya’an Hospital of Traditional Chinese Medicine, Sichuan, China.

**Keywords:** fracture, lumbar, nomogram, PKP

## Abstract

The aim was to study the independent risk factors for chronic low back pain after lumbar compression fractures undergoing percutaneous kyphoplasty (PKP), and to establish a nomogram prediction model accordingly. Data were collected from patients with lumbar compression fractures from January 2017 to December 2021 at the Affiliated Hospital of Xinjiang Medical University. Univariate and multivariate logistic regression analyses were used to determine the independent risk factors for chronic low back pain after receiving PKP for lumbar compression fractures, and the corresponding nomogram was established. Receiver operating characteristic (ROC) curves were plotted and area under the curve (AUC) was calculated, and calibration curves and decision curve analysis (DCA) were plotted to evaluate the model performance. A total of 792 patients with lumbar compression fractures were included in the study, and 188 patients had chronic postoperative low back pain, with an incidence of 23.74%. After univariate and multivariate logistic regression analysis, a total of 5 variables were identified as independent risk factors for chronic low back pain after undergoing PKP for lumbar compression fractures, namely having diabetes (OR, 1.607; 95% CI, 1.157–3.205), preoperative T value < −2.5 SD (OR, 2.697; 95% CI, 1.417–5.021), multiple lumbar fractures (OR, 1.815; 95% CI, 1.415–3.201), lumbar compression ≥ 50% (OR, 2.854; 95% CI, 1.411–6.524), and bone cement leakage (OR, 2.911; 95% CI, 1.715–6.817). The nomogram for chronic low back pain after PKP for lumbar compression fractures constructed in this study has good predictive accuracy and helps orthopedic surgeons to intervene earlier in patients at high risk of chronic low back pain after undergoing PKP for lumbar compression fractures.

## 1. Introduction

According to studies, with the increase in life expectancy and the aging of the population, lumbar compression fractures have been increasing.^[1–4]^ Lumbar compression fractures are currently one of the most common lumbar spine disorders in the elderly population.^[5,6]^ Currently, there are 3 main treatment options for lumbar compression fractures: one is conservative treatment, in which the patient requires long-term bed rest, oral pain medication, and braces; the second is a surgical treatment with traditional open surgery treatment; and the third is a minimally invasive surgical treatment with percutaneous kyphoplasty (PKP).^[7–9]^ Compared with the first 2 treatment modalities, PKP has the advantages of less trauma, shorter operative time, less intraoperative bleeding, faster recovery, earlier ambulation, and fewer postoperative complications, etc.^[10,11]^ PKP is currently the most mainstream modality for the treatment of lumbar compression fractures.^[12]^ Although there are few complications after PKP, some studies have found postoperative re-fracture of the operated vertebral body and leakage of intraoperative bone cement,^[13,14]^ but there is a lack of studies on chronic low back pain after fracture. Nomogram has been used in many fields, not only for predicting deep vein thrombosis in the lower extremities after fracture^[15]^ but also for predicting post-fracture pneumonia^[16]^ and post-fracture delirium,^[17]^ with good predictive results. We aimed to investigate the independent risk factors for chronic low back pain after lumbar compression fracture undergoing PKP and to develop a nomogram prediction model accordingly.

## 2. Information and Methods

### 2.1. Data sources

This study retrospectively analyzed the data of inpatient surgical lumbar spine compression patients from January 2017 to December 2021 at the Affiliated Hospital of Xinjiang Medical University. Relevant information about fracture patients were collected, including patient gender, age, body mass index, smoking, alcohol, hypertension, diabetes, soft tissue injury, number of fractured vertebrae, lumbar compression ratio, preoperative T value, fracture to surgery time, duration of surgery, bone cement injection volume, and bone cement leakage.

### 2.2. Inclusion and exclusion criteria

#### 2.2.1. Inclusion criteria.

First, diagnosis of lumbar compression fracture; second, patients with lumbar compression fracture admitted to hospital within 72 hours after injury; third, PKP performed after admission; fourth, complete preservation of patient information.

#### 2.2.1. Exclusion criteria.

First, patients with preoperative lumbar disorders; second, patients with combined spinal tuberculosis or spinal tumors; third, patients with previous lumbar compression fractures.

### 2.3. Clinical outcomes and definitions

Patients were followed up 6 months after surgery in the outpatient clinic or by telephone, and those with a score > 4 according to the visual analog scale (VAS) were included in the observation group and those with a VAS score ≤ 4 were included in the control group.

### 2.4. Statistical analysis

The collected data were randomly divided into a training set (70%) and a validation set (30%) according to the ratio of 7:3 in R (4.2.1) software. The differences between observation and control groups were analyzed univariately in the training set using SPSS 26.0 software, and the chi-square test was used to statistically analyze the count data. Variables screened at *P* < .05 from the univariate analysis were included in the multivariate logistic regression analysis and variables at *P* < .05 from the multivariate logistic regression analysis were identified as independent risk factors for chronic low back pain after receiving PKP for lumbar compression fractures. The screened independent risk factors were plotted in a nomogram in R software, receiver operating characteristic (ROC) curves were plotted and the area under the curve (AUC) was calculated in the training and validation sets, and calibration curves and decision curves analysis (DCA) were plotted to assess model performance.

## 3. Results

### 3.1. General information

A total of 792 patients with lumbar compression fractures were included in this study, and a total of 188 fracture patients had postoperative chronic low back pain, with an incidence of 23.74% of postoperative chronic low back pain. According to the ratio of 7:3, 556, and 236 fracture patients were randomly divided into training and validation sets.

### 3.2. Independent risk factors for postoperative chronic low back pain

In the training set, 15 variables were analyzed by univariate logistic regression analysis, and the results showed that 9 variables were potential risk factors for chronic low back pain, including age, body mass index, diabetes, soft tissue injury, preoperative T value, number of fractured vertebrae, lumbar compression rate, duration of surgery, and bone cement leakage (Table 1). Multifactorial logistic regression analysis determined that having diabetes (OR, 1.607; 95% CI, 1.157–3.205), preoperative T value < −2.5 SD (OR, 2.697; 95% CI, 1.417–5.021), multiple lumbar fractures (OR, 1.815; 95% CI, 1.415–3.201), lumbar compression ≥ 50% (OR, 2.854; 95% CI, 1.411–6.524), and bone cement leakage (OR, 2.911; 95% CI, 1.715–6.817) were independent risk factors for chronic low back pain after PKP surgery (Table 2).

**Table 1:** Univariate analysis of chronic low back pain after PKP.

Risk factors	Observation group (N = 132)	Control group (N = 424)	*P*
Age			.015
<60 yr	28 (21.21%)	137 (32.31%)	
≥60 yr	104 (78.79%)	287 (67.69%)	
Gender			.190
Male	50 (37.88%)	188 (44.34%)	
Female	82 (62.12%)	256 (55.66%)	
BMI			.043
<24	43 (32.58%)	180 (42.45%)	
≥24	89 (67.42%)	244 (57.55%)	
Smoking			.930
No	96 (72.73%)	310 (73.11%)	
Yes	36 (27.27%)	114 (26.89%)	
Alcohol			.467
No	95 (71.97%)	291 (68.63%)	
Yes	37 (28.03%)	133 (31.37%)	
Hypertension			.658
No	81 (61.36%)	251 (59.20%)	
Yes	51 (38.64%)	173 (40.80%)	
Diabetes			.001
No	69 (52.27%)	288 (67.92%)	
Yes	63 (47.73%)	136 (32.08%)	
Soft tissue injury			.034
No	117 (88.64%)	399 (94.10%)	
Yes	15 (11.36%)	25 (5.90%)	
Preoperative T value			.005
>−2.5SD	49 (37.12%)	216 (50.94%)	
≤−2.5SD	83 (62.88%)	208 (49.06%)	
Number of fractures			.047
Only one	113 (85.61%)	388 (91.51%)	
Other	19 (14.39%)	36 (8.49%)	
Lumbar compression rate			.023
<50%	64 (48.48%)	253 (59.67%)	
≥50%	68 (51.52%)	171 (40.33%)	
Fracture to surgery time			.576
<24 h	67 (50.76%)	227 (53.54%)	
≥24 h	65 (49.24%)	197 (46.46%)	
Duration of surgery			.022
<1 h	75 (56.82%)	287 (67.69%)	
≥1 h	57 (43.18%)	137 (32.31%)	
Bone cement			.394
<5 mL	78 (59.09%)	268 (63.21%)	
≥5 mL	54 (40.91%)	156 (36.79%)	
Bone cement leakage			.004
No	106 (80.30%)	381 (89.86%)	
Yes	26 (19.70%)	43 (10.14%)	

BMI = body mass index.

**Table 2:** Multifactorial analysis of chronic low back pain after PKP.

危险因素	OR	CI 95%	*P*
Age			
<60 yr	Reference	-	-
≥60 yr	2.408	0.437–13.275	.131
BMI			
<24	Reference	-	-
≥24	2.899	0.144–5.232	.487
Diabetes			
No	Reference	-	-
Yes	1.607	1.157–3.205	.017
Soft tissue injury			
No	Reference	-	-
Yes	1.104	0.369–3.308	.859
Preoperative T value			
>−2.5 SD	Reference	-	-
≤−2.5 SD	2.697	1.417–5.021	.002
Number of fractures			
Only one	Reference	-	-
Other	1.815	1.415–3.201	.011
Lumbar compression rate			
<50%	Reference	-	-
≥50%	2.854	1.411–6.524	.001
Duration of surgery			
<1 h	Reference	-	-
≥1 h	1.284	0.138–11.398	.826
Bone cement leakage			
No	Reference	-	-
Yes	2.911	1.715–6.817	<.001

BMI = body mass index.

### 3.3. Nomogram development and validation

**Figure 1.**
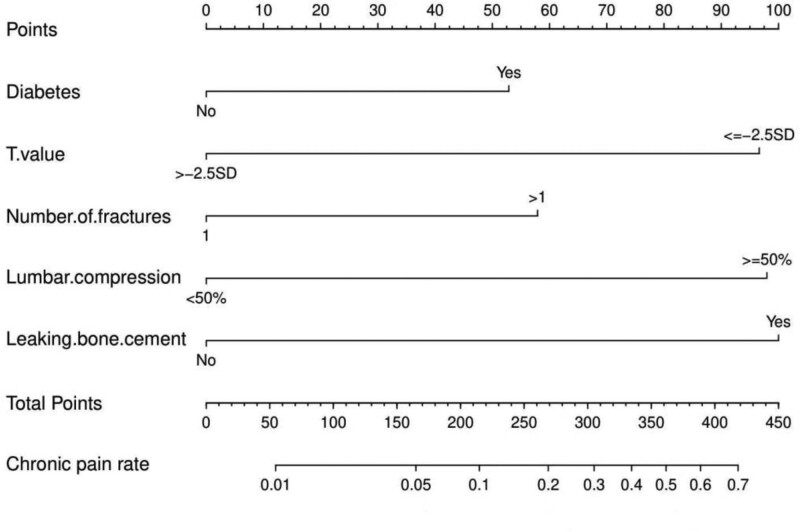
Nomogram for predicting the risk of chronic low back pain after PKP. PKP = percutaneous kyphoplasty.

**Figure 2.**
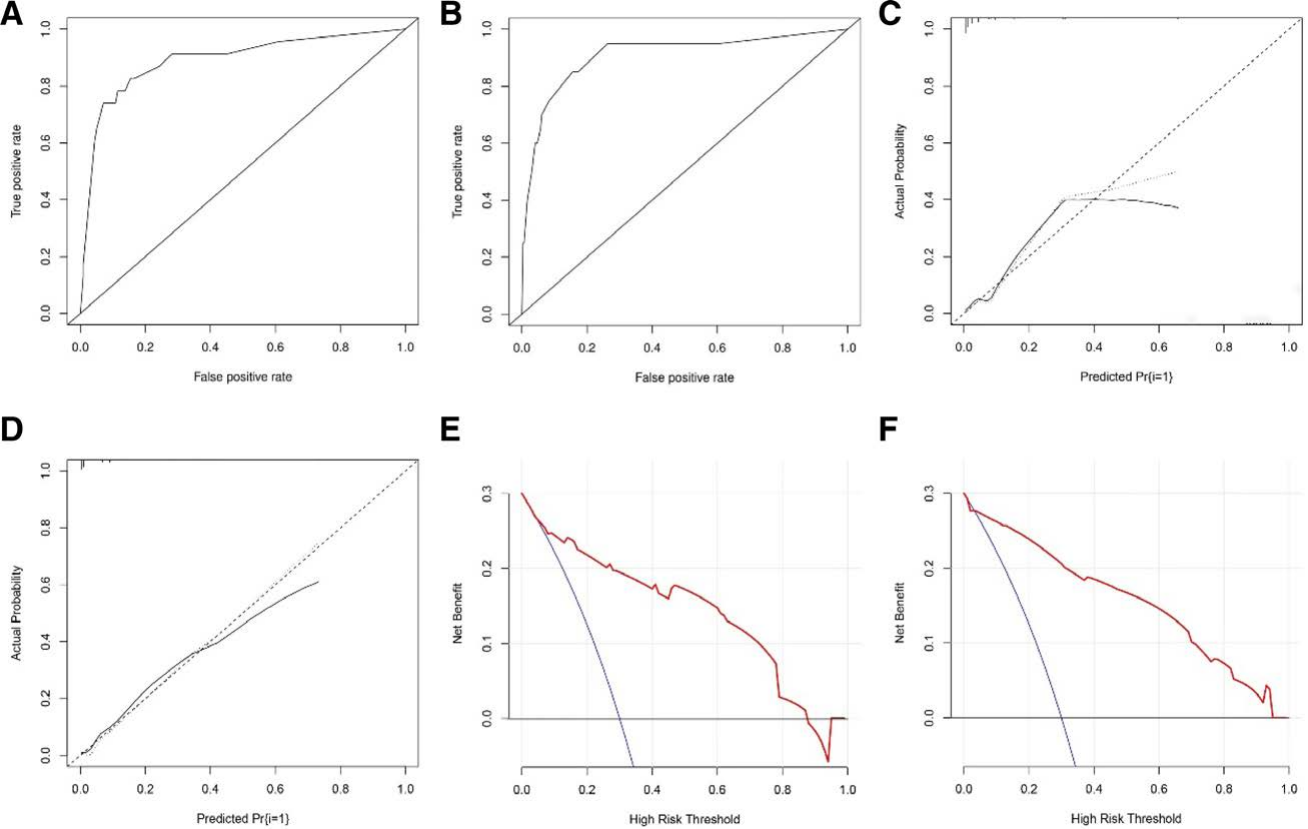
ROC curves of the nomogram for predicting chronic low back pain in the training set (A) and the validation set (B). Calibration curves of the nomogram for predicting chronic low back pain in the training set (C) and the validation set (D). DCA of the Nomogram for predicting chronic low back pain in the training set(E) and the validation set(F). DCA = decision curve analysis, ROC = receiver operating characteristic.

A nomogram was drawn using the screened independent risk factors used to predict the risk of chronic low back pain after receiving PKP for lumbar compression fractures (Fig. 1). ROC curves were then plotted for the training and validation sets, and the corresponding AUCs were calculated to be 0.891 and 0.909 (Fig. 2A and B). In addition, calibration curves were plotted, indicating that the nomogram-predicted risk agreed well with the actual risk of occurrence and had the good predictive ability (Fig. 2C and D). Also, DCA showed that the nomogram had a good predictive ability (Fig. 2E and F).

## 4. Discussion

Although PKP is currently recognized clinically as the safest and most effective method for treating lumbar compression fractures with a low rate of postoperative complications,^[10]^ some patients still have pain in the low back area after PKP treatment, which can have a serious impact on their lives.

The current study collected clinical data from patients with lumbar compression fractures over the past 5 years to develop a clinical prediction model for determining the risk of developing chronic low back pain after PKP in such patients. In this study, we used predictors that are common and easily identified in clinical practice and developed this nomogram prediction model based on the 5 independent risk factors identified, and model validation determined that the model developed in this study has good predictive power.

The results of the study showed that having diabetes is a risk factor for chronic low back pain after receiving PKP for lumbar compression fractures. Studies^[18,19]^ have shown that diabetes affects the vascular permeability of patients, causing peripheral microangiopathy, which decreases blood flow to the bone, affecting bone production and bone quality leading to increased fragility. At the same time, the microangiopathy of diabetes can cause changes in bone quality, resulting in brittle bones and fractures.^[20]^ Diabetic patients have inherently unstable blood glucose levels, which can have a very significant impact on the patient postoperative recovery.

The results of the study showed that patient with osteoporosis is a risk factor for chronic low back pain after receiving PKP for lumbar compression fractures. Studies^[21–24]^ showed that the greater the degree of vertebral osteoporosis, the lower the bone density values, and the more likely the vertebral trabeculae are to microfracture under stress, thereby stimulating the nerve endings and causing pain. Since osteoporotic microfractures can cause skeletal pain in patients, safe and effective anti-osteoporotic treatment should be administered to patients after surgery, which in turn relieves pain in the low back area.

The findings show that lumbar multiple lumbar fractures are a risk factor for chronic low back pain after receiving PKP for lumbar compression fractures. The lumbar spine carries all the weight, and an intact lumbar spine structure is the basis for function. In patients with multiple lumbar fractures, the instability of the lumbar spine increases and is less stable compared to a single lumbar fracture, which can only be partially stabilized most of the time with minimally invasive treatment and cannot maintain full stability.^[25,26]^ Postoperatively, patients often experience decreased lumbar spine stability and chronic pain in the lumbar region. For multiple lumbar fractures, traditional open surgery treatment is often recommended, as traditional open surgery treatment is more stable for stabilizing multiple lumbar fractures.^[27]^

The findings show that lumbar compression above 50% is a risk factor for chronic low back pain after receiving PKP for lumbar compression fractures. The anterior column of the vertebral body is the main bearer of axial stress in the spine and is the most vulnerable part of the entire spine to collapse. The collapse of the anterior edge of the vertebral body causes changes in the biomechanics of the vertebral body, which in turn stimulates nerve endings causing pain.^[28–30]^ In patients with lumbar fractures, the height of the vertebral body has to be restored as much as possible when using bone cement filling. For vertebrae with high compression rates, the amount of bone cement injected should be increased.

The findings show that intraoperative bone cement leakage is a risk factor for chronic low back pain after receiving PKP for lumbar compression fractures. It is generally believed that the most important pain relief mechanism of PKP is the restoration of mechanical stability of the fractured vertebral body, the diffusion of bone cement to part or the whole vertebral body through balloon propping and bone cement filling, which increases the strength of the vertebral body, fixes the vertebral microfracture, and enhances stability.^[31–33]^ However, bone cement leakage may occur when cement filling is performed, and the leaking bone cement may irritate the surrounding nerves, resulting in back pain in patients. Therefore, when performing bone cement infusion, the speed of bone cement infusion should be controlled, and the amount of bone cement should be controlled.

However, there are shortcomings in this study. First, because this was a retrospective study, some unavoidable error arises. Second, this is a single-center study conducted at a tertiary referral trauma center, and there is bias in the selection of patients due to more severe fractures or more complex conditions in the admitted patients. Third, this is a risk prediction model developed in a single center, and therefore its validity needs to be validated in further multicenter studies.

## 5. Conclusion

The nomogram for chronic low back pain after PKP for lumbar compression fractures constructed in this study has good predictive accuracy and helps orthopedic surgeons to intervene earlier in patients at high risk of chronic low back pain after undergoing PKP for lumbar compression fractures.

## Author contributions

**Conceptualization:** Guang-hua Deng.

**Data curation:** Guang-hua Deng.

**Formal analysis:** Guang-hua Deng.

**Funding acquisition:** Guang-hua Deng.

**Investigation:** Guang-hua Deng.

**Methodology:** Guang-hua Deng.

**Project administration:** Guang-hua Deng.

**Resources:** Guang-hua Deng.

**Software:** Guang-hua Deng.

**Supervision:** Guang-hua Deng.

**Validation:** Guang-hua Deng.

**Visualization:** Guang-hua Deng.

**Writing – original draft:** Guang-hua Deng.

**Writing – review & editing:** Guang-hua Deng.

## References

[1] Bouyer B Vassal M Zairi F. Surgery in vertebral fracture: epidemiology and functional and radiological results in a prospective series of 518 patients at 1 year’s follow-up. Orthop Traumatol Surg Res. 2015;101:11–5. 25596983 10.1016/j.otsr.2014.11.012

[2] Falavigna A Righesso O Guarise da Silva P. Epidemiology and management of spinal trauma in children and adolescents <18 Years Old. World Neurosurg. 2018;110:e479–83. 29146435 10.1016/j.wneu.2017.11.021

[3] Fryhofer GW Smith HE. Return to play for cervical and lumbar spine conditions. Clin Sports Med. 2021;40:555–69. 34051946 10.1016/j.csm.2021.04.002

[4] Graham P. Lumbar compression fracture. Orthop Nurs. 2021;40:104–6. 33756539 10.1097/NOR.0000000000000750

[5] Shin CS Kim MJ Shim SM. The prevalence and risk factors of vertebral fractures in Korea. J Bone Miner Metab. 2012;30:183–92. 21773702 10.1007/s00774-011-0300-x

[6] Park GR Kim HS Kim YT. Waist circumference and the risk of lumbar and femur fractures: a nationwide population-based cohort study. Eur Rev Med Pharmacol Sci. 2021;25:1198–205. 33629289 10.26355/eurrev_202102_24822

[7] Wu X Ma W Du H. A review of current treatment of lumbar posterior ring apophysis fracture with lumbar disc herniation. Eur Spine J. 2013;22:475–88. 23179980 10.1007/s00586-012-2580-9 PMC3585633

[8] Chen F Kang Y Li H. Treatment of lumbar split fracture-dislocation with short-segment or long-segment posterior fixation and anterior fusion. Clin Spine Surg. 2017;30:E310–6. 28323717 10.1097/BSD.0000000000000182

[9] Bork H Simmel S Böhle E. [Rehabilitation after traumatic fracture of thoracic and lumbar spine]. Z Orthop Unfall. 2018;156:533–40. 29775977 10.1055/a-0591-6712

[10] Liu H Zhou Q Shao X. Percutaneous kyphoplasty in patients with severe osteoporotic vertebral compression fracture with and without intravertebral cleft: a retrospective comparative study. Int J Gen Med. 2022;15:6199–209. 35880137 10.2147/IJGM.S369840 PMC9307916

[11] Zhu D Hu J Wang L. A comparison between modified unilateral extrapedicular and bilateral transpedicular percutaneous kyphoplasty in the treatment of lumbar osteoporotic vertebral compression fracture. World Neurosurg. 2022;166:e99–e108. 35779757 10.1016/j.wneu.2022.06.115

[12] Jin J Shen W. Long-term therapeutic effect of percutaneous kyphoplasty combined with & without back muscle rehabilitation exercise in elderly patients A comparative study. Pak J Med Sci. 2022;38:1595–600. 35991245 10.12669/pjms.38.6.5873 PMC9378420

[13] Yin P Li Z Zhu S. The treatment of osteoporotic thoraco-lumbar burst fractures by unilateral percutaneous kyphoplasty: a prospective observation study. Eur J Pain. 2020;24:659–64. 31782863 10.1002/ejp.1516

[14] Dai C Liang G Zhang Y. Risk factors of vertebral re-fracture after PVP or PKP for osteoporotic vertebral compression fractures, especially in Eastern Asia: a systematic review and meta-analysis. J Orthop Surg Res. 2022;17:161. 35279177 10.1186/s13018-022-03038-z PMC8917756

[15] Lin Z Mi B Liu X. Nomogram for predicting deep venous thrombosis in lower extremity fractures. Biomed Res Int. 2021;2021:9930524. 34258284 10.1155/2021/9930524 PMC8245242

[16] Zhang X Shen Z-L Duan X-Z. Postoperative pneumonia in geriatric patients with a hip fracture: incidence, risk factors and a predictive nomogram. Geriatr Orthop Surg Rehabil. 2022;13:21514593221083824. 10.1177/21514593221083824 PMC8949772 35340623

[17] Yang Y Wang T Guo H. Development and validation of a nomogram for predicting postoperative delirium in patients with elderly hip fracture based on data collected on admission. Front Aging Neurosci. 2022;14:914002. 35783136 10.3389/fnagi.2022.914002 PMC9243358

[18] Shah VN Harrall KK Shah CS. Bone mineral density at femoral neck and lumbar spine in adults with type 1 diabetes: a meta-analysis and review of the literature. Osteoporos Int. 2017;28:2601–10. 28580510 10.1007/s00198-017-4097-x

[19] Compston J. Type 2 diabetes mellitus and bone. J Intern Med. 2018;283:140–53. 29265670 10.1111/joim.12725

[20] Leslie WD Aubry-Rozier B Lamy O. TBS (trabecular bone score) and diabetes-related fracture risk. J Clin Endocrinol Metab. 2013;98:602–9. 23341489 10.1210/jc.2012-3118

[21] Bouxsein ML Eastell R Lui L-Y. Change in bone density and reduction in fracture risk: a meta-regression of published trials. J Bone Miner Res. 2019;34:632–42. 30674078 10.1002/jbmr.3641

[22] Johnston CB Dagar M. Osteoporosis in older adults. Med Clin North Am. 2020;104:873–84. 32773051 10.1016/j.mcna.2020.06.004

[23] Urquiaga M Saag KG. Risk for osteoporosis and fracture with glucocorticoids. Best Pract Res Clin Rheumatol. 2022;36:101793. 36347775 10.1016/j.berh.2022.101793

[24] Wáng YXJ Xiao BH. Estimations of bone mineral density defined osteoporosis prevalence and cutpoint T-score for defining osteoporosis among older Chinese population: a framework based on relative fragility fracture risks. Quant Imaging Med Surg. 2022;12:4346–60. 36060578 10.21037/qims-22-281 PMC9403581

[25] Feichtinger X Kocijan R Mittermayr R. Fracture patterns in patients with multiple fractures: the probability of multiple fractures and the most frequently associated regions. Eur J Trauma Emerg Surg. 2020;46:1151–8. 30747275 10.1007/s00068-019-01087-4

[26] Cozadd AJ Schroder LK Switzer JA. Fracture risk assessment: an update. J Bone Joint Surg Am. 2021;103:1238–46. 33830957 10.2106/JBJS.20.01071

[27] Chalhoub D Orwoll ES Cawthon PM. Areal and volumetric bone mineral density and risk of multiple types of fracture in older men. Bone. 2016;92:100–6. 27554426 10.1016/j.bone.2016.08.014 PMC5056840

[28] Wade KR Robertson PA Thambyah A. How healthy discs herniate: a biomechanical and microstructural study investigating the combined effects of compression rate and flexion. Spine (Phila Pa 1976) 2014;39:1018–28. 24503692 10.1097/BRS.0000000000000262

[29] Stemper BD Yoganandan N Baisden JL. Rate-dependent fracture characteristics of lumbar vertebral bodies. J Mech Behav Biomed Mater. 2015;41:271–9. 25154535 10.1016/j.jmbbm.2014.07.035

[30] Petitt JC Desai A Kashkoush A. Failure of conservatively managed traumatic vertebral compression fractures: a systematic review. World Neurosurg 2022;165:81–8. 35724881 10.1016/j.wneu.2022.06.053

[31] Zhu SY Zhong Z-M Wu Q. Risk factors for bone cement leakage in percutaneous vertebroplasty: a retrospective study of four hundred and eighty five patients. Int Orthop. 2016;40:1205–10. 26753843 10.1007/s00264-015-3102-2

[32] Liu T Li Z Su Q. Cement leakage in osteoporotic vertebral compression fractures with cortical defect using high-viscosity bone cement during unilateral percutaneous kyphoplasty surgery. Medicine (Baltim). 2017;96:e7216. 10.1097/MD.0000000000007216 PMC5484220 28640112

[33] Zhang S Wang GJ Wang Q. A mysterious risk factor for bone cement leakage into the spinal canal through the Batson vein during percutaneous kyphoplasty: a case control study. BMC Musculoskelet Disord. 2019;20:423. 31510985 10.1186/s12891-019-2807-6 PMC6739913

